# Discrimination Against Older Adults and Related Behaviors Among Nurses: A Cross-sectional Study

**DOI:** 10.1097/jnr.0000000000000743

**Published:** 2026-05-08

**Authors:** Chih-Yu WANG, Cheng-Hua NI, Ching-Wen CHIU, Ruey CHEN, I-Hui CHEN, Megan F. LIU, Yeu-Hui CHUANG

**Affiliations:** 1 College of Nursing, The Ohio State University, Columbus, OH, USA; 2 School of Nursing, College of Nursing, Taipei Medical University, Taipei, Taiwan; 3 Department of Nursing, Wan Fang Hospital, Taipei Medical University, Taipei, Taiwan; 4 Center for Management and Development, Taipei Medical University, Taipei, Taiwan; 5 Department of Nursing, Shuang-Ho Hospital, Taipei Medical University, New Taipei City, Taiwan; 6 Post-Baccalaureate Program in Nursing, College of Nursing, Taipei Medical University, Taipei, Taiwan; 7 School of Gerontology and Long-Term Care, College of Nursing, Taipei Medical University, Taipei, Taiwan; 8 College of Interdisciplinary Studies, Taipei Medical University, Taipei, Taiwan; 9 Research Center in Nursing Clinical Practice, Wan Fang Hospital, Taipei Medical University, Taipei, Taiwan

**Keywords:** nurse, older adult, ageism, stereotype, discrimination

## Abstract

**Background:**

With the population of older adults growing, nurses are increasingly likely to care for older patients. Ageist attitudes and behaviors in nurses may affect care quality, patient health, and care willingness. Ageism, referring to prejudice, stereotyping, or discrimination against individuals or groups based on their age, has been widely studied in the literature, primarily in non–East Asian countries, and few studies have been designed to investigate ageism and ageist behaviors among hospital nurses in Taiwan.

**Purpose:**

This study was designed to investigate ageism and ageist behaviors among hospital nurses and identify the factors associated with ageist behaviors.

**Methods:**

A cross-sectional approach was used, and 359 nurses in Taiwan were enrolled as participants. Data were collected using a structured questionnaire comprising sociodemographic characteristics, the Fraboni Scale of Ageism (FSA), and Relating to Older People Evaluation (ROPE).

**Results:**

The mean FSA scale score was 68.77 ± 8.83 and the mean proportion of ROPE was 0.39 ± 0.13, with 0.51 ± 0.16 for positive ageist behaviors and 0.34 ± 0.15 for negative ageist behaviors. Age, working unit, having taken age-related courses at school, and aging anxiety were all shown to relate significantly to ageist behaviors. In addition, a significant association was found between ageism and ageist behaviors. Predictors of ageist behaviors include age, working unit, having taken age-related courses at school, and ageism.

**Conclusions:**

The findings of this study indicate discriminatory attitudes and behaviors toward older adults exist among hospital nurses and that nurses who are older, work in operating rooms or critical care units, have not taken age-related courses in school, or have higher levels of ageism are at higher relative risk of exhibiting ageist behaviors. The findings of this study may be used to guide nursing managers in developing tailored interventions for nurses across different age groups and work units to effectively prevent or mitigate ageist behaviors. Furthermore, nursing educators may use this information to design age-related courses to enhance student awareness of ageist behaviors.

## Introduction

Projections indicate that people aged over 65 will surpass the number of children aged under 18 by the late 2070s, reaching 2.2 billion (United Nations, 2024). Taiwan officially became an aging society (with 14% of the population ≥ 65 years old) in 2018. This percentage rose to 20.22% by the end of February 2026 (Ministry of the Interior, 2026). Taiwan has entered a super-aged society (when 20% of the population is expected to be ≥ 65 years old) and by 2070 is expected to have a population in which 46.54% are 65 years or older (National Development Council, 2025). Older adults are frequently perceived as less valuable, unproductive, and dependent due to frailty resulting from age-related declines in physical and mental capacities (Jönson & Annika, 2019). Ageism, referring to prejudice, stereotyping, or discrimination against individuals or groups based on their age, has emerged as a significant global issue, spanning various countries and cultural contexts (World Health Organization, 2021).

Butler (1969) proposed that ageism may be understood as a systematic process of stereotyping and discriminating against people based on age, the mechanisms of which are similar to how racism and sexism operate, based, respectively, on skin color and sex. Ageism encompasses stereotypes, prejudices, and discrimination that influence each other. Stereotypes involve cognitive structures that incorporate misconceptions about older adults, perceiving them to be frail and dependent. Prejudices include ageist attitudes and negative emotions towards older adults and aging. Discrimination relates to behaviors that involve inappropriate actions based on age (World Health Organization, 2021), which can be positive or negative (Palmore, 1999). Positive ageist behaviors such as elderspeak, which uses exaggerated prosody or patronizing language, may seem benevolent or courteous but are rooted in bias (Shaw & Gordon, 2021) and often go unnoticed because they appear respectful toward older adults. However, they may adversely affect the self-esteem and social standing of older individuals by exacerbating their self-perceptions of aging (Marquet et al., 2019). Negative ageist behaviors include mocking, ignoring, or abusing older adults (Palmore, 1999).

Ageism seriously impacts older adult physical health in aspects such as chronic conditions, functional impairments, hospitalizations (Chang et al., 2020), and mental health (depression, anxiety, and stress; Bodner et al., 2018). Chang et al. (2020) found that negative self-perceptions of aging among older adults were a predictor of engaging in risky health behaviors, a shorter lifespan, and poorer quality of life.

The results of previous studies support that ageism affects the willingness of health care professionals and nursing students to provide care to older patients (Even et al., 2020). In health care settings, negative attitudes toward older adults among nurses may discourage nursing students from caring for older patients in their future careers (Attafuah et al., 2022). A recent literature review revealed nurses may transmit their stereotypes and biases about older adults unconsciously to the next shift, fostering the spread of ageism (Van, 2020). Ageism displayed by health care professionals can compromise patient care quality and health, leading to the neglect of older patients during caregiving (Rababa et al., 2020). Therefore, it is crucial that nurses be aware of their ageism and ageist behaviors, as they are the primary health care providers responsible for caring holistically for the physical, psychological, social, and spiritual needs of older patients.

In prior studies using the Fraboni Scale of Ageism (FSA) to measure ageism (Fraboni et al., 1990), doctors and nurses in Israel were found to have a weak to moderate level (Kolushev et al., 2021), while nurses in Russia were found to have a moderate level (Punchik et al., 2021). A study conducted in Turkey identified a high level of ageist attitudes among hospital nurses (80.62 ± 13.92; FSA cutoff value = 78; Ozel & Kutlu, 2021). In terms of measuring ageist behaviors among nurses, a Korean study using the Relating to Older People Evaluation (ROPE) scale found frequencies of positive and negative ageist behaviors of, respectively, 0.36 and 0.26 among physicians, 0.45 and 0.27 among nurses, and 0.41 and 0.39 among medical students (Lee et al., 2020). Moreover, the findings of other studies in which the ROPE scale was used identified the frequency of positive ageist behaviors as higher than negative ageist behaviors among nurses, nursing students, and health care students (Even et al., 2020; Rababa et al., 2020; Rababa et al., 2021).

Multiple factors of influence on ageism and ageist behaviors among health care workers have been identified in prior studies. Ozel and Kutlu (2021) found that health care workers with lower levels of education or who were dissatisfied with their jobs showed higher levels of ageism. Rababa and Hammouri (2021) identified marital status, educational level, type of hospital, and cohabitating with older adults as significant factors of influence on positive ageist behaviors. In addition, ageist behaviors were shown to be significantly associated with the ratio of older patients treated and having taken a geriatrics course (Lee et al., 2020). Furthermore, a positive correlation has been reported between ageism and ageist behaviors (Rababa et al., 2021) and a negative correlation has been reported between ageism and positive ageist behaviors (Even et al., 2020; Rababa et al., 2021).

Most ageism studies in the literature were conducted in non-East Asian countries, with few studies investigating the factors related to ageist behaviors. Thus, more research is required to elucidate the issue of ageism in nurses and the potential effects of culture on ageist behaviors. This study was developed to investigate ageism and ageist behaviors among hospital nurses in Taiwan and to identify factors significantly associated with ageist behaviors. The research hypotheses were: (1) sociodemographic characteristics and ageism are predictors of ageist behavior in nurses, and (2) ageism in nurses relates to ageist behaviors.

## Methods

### Research Design and Participants

This cross-sectional research study was conducted in three hospitals in northern Taiwan. Registered or licensed practical nurses who were at least 20 years old with a minimum of three months of clinical experience were eligible to participate. Part-time nurses, nurse practitioners, nursing supervisors, and directors were excluded.

The minimum sample size was determined to be 270 using G-power analysis, considering a 0.12 effect size (Kolushev et al., 2021), a type I error of 0.05, a power level of 0.90, 19 predictor variables in the linear multiple regression analysis, and a response rate of 73% (Feng et al., 2020). Three hundred sixty-five participants were enrolled, six of whom were excluded from analysis due to incomplete questionnaires (response rate: 98.4%). A two-level stratified sampling approach was used in each hospital to recruit nurses. First, the sample size for each hospital was determined. Second, questionnaires were distributed to each ward within each hospital based on the proportion of total nurses in each ward.

### Instruments

#### Sociodemographic Characteristics

This questionnaire, developed based on a literature review, collected data on sociodemographic characteristics, including age, sex, religious beliefs, educational level, marital status, length of nursing experience (years), type of hospital, working unit, position, clinical ladder level, job satisfaction (on a scale of 1 [*low*] to 5 [*high*]), frequency of contact with older patients, cohabitation with older adults, having attended age-related courses or training as in-service education, having taken age-related courses at school, willingness to care for older adults (on a scale of 1 [*low*] to 5 [*high*]), level of aging anxiety (on a scale of 1 [*low*] to 5 [*high*]), and level of death anxiety (on a scale of 1 [low] to 5 [high]).

#### Fraboni Scale of Ageism

Ageism was measured using the FSA, which contains 29 items and is scored on a 4-point Likert Scale from 1 (*strongly disagree*) to 4 (*strongly agree*; Fraboni et al., 1990). Items 8, 12, 14, 21, 22, 23, and 24 are positive statements and were thus reverse-scored. The total possible scale score ranges from 29 to 116, with higher scores indicating higher levels of ageism. Because no Chinese version of the FSA scale is currently available, it was translated for this study based on Brislin’s translation model (Jones et al., 2001). The content validity of the Chinese version of the FSA was assessed for stability by five experts, with a content validity index (CVI) for each item ranging from .80 to 1.0, and a total scale CVI of .99. The Cronbach’s alpha reliability was .86 both in the original study (Fraboni et al., 1990) and in this study.

#### Relating to Older People Evaluation

The frequency and types of ageist behaviors were measured using the Chinese version of ROPE (Liu & Liu, 2019), which was adapted from the original English-language version developed by Cherry and Palmore (2008). The ROPE scale contains 20 items, including six positive (1, 3, 5, 7, 9, and 15) and 14 negative ageist behaviors. Each item presents three options: “*never*” (0 points), “*sometimes*” (1 point), and “*often*” (2 points). In terms of total possible scores, the total scale ranges from 0 to 40, the positive ageist behaviors score ranges from 0 to 12, and the negative ageist behaviors score ranges from 0 to 28. The frequency of ageist behaviors among nurses was expressed as a proportion by summing the item scores and dividing by the maximum score of 40. The frequency of positive ageist behaviors was determined by summing and dividing the six positive items by the maximum score of 12, while the frequency of negative ageist behaviors was calculated similarly using the 14 negative items, with higher proportions indicating a higher frequency of ageist behaviors. The Cronbach’s alpha reliability was .70, with .57 for positive ageist behaviors and .72 for negative ageist behaviors in the original study (Cherry & Palmore, 2008), and was .82 in this study, with .60 for positive ageist behaviors and .80 for negative ageist behaviors.

### Data Collection

After obtaining ethical approval, the researchers met with the nurse supervisor or vice director of each participating hospital to discuss the study. A presentation was conducted during the nursing managers’ meeting to provide an overview of the study. Moreover, researchers verbally explained the research purpose, procedure, and related rights to nurses during staff meetings. Eligible nurses who agreed to participate completed the questionnaires within an average of 15–20 minutes, returning them in a sealed box located at each nursing station. Data were collected from November 2022 to January 2023.

### Data Analysis

Before data analysis, the researchers checked the accuracy and integrity of the raw data twice. Data were analyzed using SPSS Version 18 (SPSS, Chicago, IL, USA), with *p* < .05 used to indicate statistical significance for all statistical tests. Percentage, mean, *SD*, Pearson correlations, *t* test, and one-way analysis of variance were all used to analyze the data, and multiple linear regression was employed to identify the predictors of ageist behaviors.

### Ethical Considerations

Ethical approval for this study was obtained from Taipei Medical University - Joint Institutional Review Board (approval number: N202210081). Rather than signing informed consent, returning the questionnaire was used to indicate agreement to participate.

## Results

### Sociodemographic Characteristics

The average age of the 359 participants was 31.22 (*SD* = 7.64; range = 21~61) years. Most were female (*n* = 341, 95.0%), had religious beliefs (*n* = 252, 70.2%), held at least a bachelor’s degree (*n* = 288, 80.2%), and were single (*n* = 275, 76.6%). Their average nursing experience was 8.63 (*SD* = 6.86) years, with 66.0% working in a regional hospital. Most worked in medical or surgical units (MSUs; 41.8%), followed by critical care units (CCUs; 24.5%), outpatient departments (OPDs; 10.0%), operating rooms (ORs; 10.3%), obstetrics and gynecology and pediatrics units (OGPUs; 10.9%), and psychiatric units (PUs; 2.5%). Registered nurses (RNs) comprised 96.4%, with 54.0% at level 2 on the clinical ladder. The average job satisfaction score was 3.54 (*SD* = 0.75). Participants spent an average of 26.25 (*SD* = 16.92) hours/week working with older adults, and 84.4% did not cohabitate with an older adult. Regarding courses and training, 74.4% had received age-related courses or training as in-service education, and 91.1% had taken age-related courses at school. The mean willingness to care for older adults score was 3.55 (*SD* = 0.77), while those for aging anxiety and death anxiety were 2.27 (*SD* = 1.03) and 1.98 (*SD* = 1.01), respectively (Table 1).

**Table 1:** Sociodemographic Characteristics of Participants (*N* = 359)

Characteristic	*n* (%)
Age (years, mean and *SD*)	31.22 (7.64)
Range	21–61
Sex	
Male	18 (5.0)
Female	341 (95.0)
Religious beliefs	
None	107 (29.8)
Yes	252 (70.2)
Educational level	
Associate degree	71 (19.8)
Bachelor’s degree or above	288 (80.2)
Marital status	
Single	275 (76.6)
Married	84 (23.4)
Length of nursing experiences (years, mean and *SD*)	8.63 (6.86)
Range	0.20–41.00
Type of hospital	
Medical center	122 (34.0)
Regional hospital	237 (66.0)
Working unit (hospital)	
Medical and surgical unit	150 (41.8)
Critical care unit	88 (24.5)
Outpatient department	36 (10.0)
Operating room	37 (10.3)
Obstetrics and gynecology pediatrics units	39 (10.9)
Psychiatry unit	9 (2.5)
Position	
Licensed practical nurses	13 (3.6)
Registered nurse	346 (96.4)
Clinical ladder level	
N	39 (10.9)
N1	56 (15.6)
N2	194 (54.0)
N3 or above	70 (19.5)
Job satisfaction (mean and *SD*)	3.54 (0.75)
Range	1–5
Frequency of contact with older patients(hours per week; mean and *SD*)	26.25 (16.92)
Range	0–70
Cohabitation with older adults	
No	303 (84.4)
Yes	56 (15.6)
Have taken age-related courses or training as in-service education	
No	92 (25.6)
Yes	267 (74.4)
Have taken age-related courses at school	
No	32 (8.9)
Yes	327 (91.1)
Willing to care for older adults (mean and *SD*)	3.55 (0.77)
Range	1–5
Aging anxiety (mean and *SD*)	2.27 (1.03)
Range	1–5
Death anxiety (mean and *SD*)	1.98 (1.01)
Range	1–5

*Note.* The Taiwan Nurses Association defines clinical ladder levels as follows: N1: Completed one year of clinical work and N1 professional training, possessing basic care capabilities; N2: Completed two years of clinical work and N2 professional training, participating in the care of critically ill patients; N3: Completed three years of clinical work and N3 professional training, demonstrating comprehensive nursing, teaching, and quality improvement abilities; N4: Completed four years of clinical work and N4 professional training, exhibiting comprehensive nursing, teaching, administrative, and quality improvement skills.

### Nurses’ Ageism and Ageist Behaviors

The mean FSA scale score was 68.77 (*SD* = 8.8), while the average FSA item score was 2.37 (*SD* = 0.30). The mean proportion of ROPE was .39 (*SD* = 0.13). The frequency of positive ageist behaviors was .51 (*SD* = 0.16), while that for negative ageist behaviors was 0.34 (*SD* = 0.15).

### Associations Between Sociodemographic Characteristics and Ageist Behaviors

Ageist behaviors were found to be significantly associated with age (*r* = .15, *p* = .004), working unit (*F* = 2.35, *p* = .041), having taken age-related courses at school (*t* = 2.59, *p* = .010), and aging anxiety (*r* = .11, *p* = .043). In addition, positive ageist behaviors were shown to significantly associate with age (*r* = .13, *p* = .018), job satisfaction (*r* = .15, *p* = .006), and willingness to care for older adults (*r* = .20, *p* < .001). Furthermore, negative ageist behaviors were shown to significantly associate with age (*r* = .14, *p* = .009), working unit (*F* = 2.67, *p* = .022), position (*t* = 2.29, *p* = .022), having taken age-related courses at school (*t* = 2.64, *p* = .009), and aging anxiety (*r* = .11, *p* = .035; Tables 2 and 3). The results of the post hoc analysis identified higher frequencies of ageist behaviors and negative ageist behaviors in CCU and OR nurses than in MSU nurses.

**Table 2:** Differences in Ageist Behaviors by Sociodemographic Characteristics

Characteristic	All Ageist Behaviors		Positive Ageist Behaviors		Negative Ageist Behaviors	
	*M ± SD*	*t/F(p)* Scheffe’s	*M ± SD*	*t/F(p)* Scheffe’s	*M ± SD*	*t/F(p)* Scheffe’s
Sex		−0.94 (.350)		−1.72 (.085)		−0.40 (.692)
Male	.36 ±.13		.44 ±.17		.32 ±.13	
Female	.39 ±.13		.51 ±.16		.34 ±.15	
Religious beliefs		−0.94 (.346)		−1.06 (.288)		−0.71 (.478)
None	.38 ±.14		.49 ±.16		.33 ±.15	
Yes	.39 ±.13		.51 ±.16		.34 ±.15	
Educational level		−0.28 (.781)		1.34 (.180)		0.43 (.330)
Associate degree	.38 ±.13		.53 ±.17		.32 ±.14	
Bachelor’s degree or above	.39 ±.13		.50 ±.16		.34 ±.15	
Marital status		−0.76 (.449)		−0.22 (.829)		−0.87 (.387)
Single	.38 ±.13		.51 ±.16		.33 ±.15	
Married	.40 ±.14		.51 ±.16		.35 ±.16	
Type of hospital		1.12 (.262)		0.43 (.669)		1.23 (.218)
Medical center	.40 ±.15		.51 ±.16		.35 ±.16	
Regional hospital	.38 ±.13		.51 ±.16		.33 ±.14	
Working unit (hospital)		2.35 (.041*)		1.33 (.250)		2.67 (.022*)
①MSU	.36 ±.12		.50 ±.16		.31 ±.13	
②CCU	.40 ±.14	②>①	.52 ±.16		.35 ±.16	②>①
③OPD	.38 ±.17	④>①	.50 ±.20		.33 ±.18	④>①
④OR	.43 ±.15		.52 ±.16		.39 ±.17	
⑤OGPU	.40 ±.13		.52 ±.17		.35 ±.14	
⑥PU	.43 ±.17		.63 ±.13		.35 ±.23	
Position		1.54 (.150)		1.84 (.066)		2.29 (.022*)
Licensed practical nurse	.48 ±.22		.59 ±.19		.43 ±.23	
Registered nurse	.38 ±.13		.51 ±.16		.33 ±.15	
Clinical ladder level		0.62 (.606)		0.76 (.517)		0.36 (.785)
N	.37 ±.14		.49 ±.19		.32 ±.16	
N1	.38 ±.13		.50 ±.17		.33 ±.14	
N2	.40 ±.13		.52 ±.15		.34 ±.15	
N3 or above	.38 ±.14		.50 ±.17		.33 ±.15	
Cohabitation with older adults		−1.16 (.248)		−1.07 (.288)		−0.98 (.327)
No	.38 ±.13		.50 ±.16		.33 ±.15	
Yes	.41 ±.14		.53 ±.18		.35 ±.16	
Have taken age-related courses or training as in-service education		−0.52 (.606)		−1.59 (.113)		0.07 (.941)
No	.38 ±.15		.49 ±.16		.34 ±.16	
Yes	.39 ±.13		.52 ±.16		.33 ±.15	
Have taken age-related courses at school		2.59 (.010*)		1.40 (.162)		2.64 (.009**)
No	.45 ±.19		.55 ±.19		.40 ±.20	
Yes	.38 ±.13		.50 ±.16		.33 ±.14	

*Note.* MSU = medical or surgical units; CCU = critical care unit; OPD = outpatient department; OR = operating room; OGPU = obstetrics and gynecology, and pediatrics unit; PU = psychiatric unit.

*p < .05. **p < .001.

**Table 3:** Correlations Among Sociodemographic Characteristics, Ageism, and Ageist Behaviors

Variable	Age	Length of Nursing Experience (Years)	Job Satisfaction	Frequency of Contact with Older Patients	Willingness to Care for Older Adults	Aging Anxiety	Death Anxiety	Ageism	Ageist Behaviors	Positive Ageist Behaviors	Negative Ageist Behaviors
Age	1	.92**	.03	-.19**	-.01	.15**	.10	.14**	.15**	.13*	.14**
Length of nursing experiences (years)	.92**	1	.03	-.16**	.04	.15**	.11*	.11*	.08	.09	.07
Job satisfaction	.03	.03	1	-.10	.21**	-.06	-.01	-.10	.04	.15**	-.01
Frequency of contact with older patients	-.19**	-.16**	-.10	1	.16**	-.01	-.08	-.14*	-.07	-.01	-.08
Willingness to care for older adults	-.01	.04	.21**	.16**	1	-.07	.01	-.31***	-.01	.20***	-.10
Aging anxiety	.15**	.15**	-.06	-.01	-.07	1	.52**	.21***	.11*	.05	.11*
Death anxiety	.10	.11*	-.01	-.08	.01	.52*	1	.14*	.10	.08	.09
Ageism	.14**	.11*	-.10	-.14*	-.31***	.21***	.14*	1	.31***	-.06	.42***
Ageist behaviors	.15**	.08	.04	-.07	-.01	.11*	.10	.31***	1	.71***	.95***
Positive ageist behaviors	.13*	.09	.15**	-.01	.20***	.05	.08	-.06	.71***	1	.44***
Negative ageist behaviors	.14**	.07	-.01	-.08	-.10	.11*	.09	.42***	.95***	.44***	1

*p < .05.**p < .01.***p < .001.

### Associations Among Ageism, Ageist Behaviors, Positive Ageist Behaviors, and Negative Ageist Behaviors

As seen in Table 3, positive correlations were respectively found between ageism and ageist behaviors (*r* = .31, *p* < .001) and between ageism and negative ageist behaviors (*r* = .42, *p* < .001). However, no significant correlation was identified between ageism and positive ageist behaviors (*r* = -.06, *p* = .297).

### Predictors of Ageist Behaviors in Nurses

The variables identified in the above analysis as significantly associated with ageist behaviors, including age, having taken age-related courses at school, working unit, aging anxiety, and ageism, were entered into the multiple linear regression model (Table 4). The variables identified by the model as predictors of ageist behaviors included age, working unit, having taken age-related courses at school, and ageism, which collectively predicted 11.9% of the variance in ageist behaviors in the sample (*F* = 6.364, *p* < .001).

**Table 4:** Multiple Linear Regression Model for Predictors of Ageist Behaviors

Variable	*B*	*SE*	*t*	*p*	Multicollinearity	
					Tolerance	VIF
Constant	.066	.063	1.046	.296		
Age	.002	.001	2.033	.043*	.91	1.10
Working unit (ref: MSU)						
CCU	.038	.017	2.265	.024*	.83	1.20
OPD	-.013	.024	−0.533	.595	.85	1.18
OR	.041	.024	1.732	.084	.86	1.17
OGPU	.020	.023	0.868	.386	.87	1.15
PU	.060	.043	1.377	.169	.97	1.04
Have taken age-related courses at school (ref: no)	-.050	.024	−2.115	.035*	.97	1.03
Aging anxiety	.003	.007	0.508	.612	.92	1.09
Ageism	.004	.001	5.229	<.001***	.91	1.10

*Note. R* ^2^ = .141, adjusted *R* ^2^ = .119, *F* = 6.364, *p* < .001.

VIF = variance inflation factor; ref = reference group; MSU = medical or surgical unit; CCU = critical care unit; OPD = outpatient department; OR = operating room; OGPU = obstetrics and gynecology, and pediatrics unit; PU = psychiatric unit.

*p < .05. ***p < .001.

## Discussion

The findings of this study identified ageism as present among the hospital nurses in the sample, with an average total score of 68.77 on the FSA scale. This score is similar to the 69.50 reported in a Jordanian study (Rababa et al., 2020), lower than the 79.33 reported in a Turkish study (Ozel & Kutlu, 2021), and higher than the 64.80 reported in a Korean study (Lee et al., 2020). Comparing average item scores on the FSA scale, the nurses in this study had a lower level of ageism (2.37) than their counterparts in Israel (2.80; Kolushev et al., 2021) and Korea (2.63; Hwang & Kim, 2021). Although the nurses in this study demonstrated a moderate level of ageism, their FSA scores were lower than those reported in most previous studies on hospital nurses. One possible reason for this may be the deep influence of Confucian principles, which emphasize filial piety and ethical hierarchies, on Taiwanese culture, strengthening the idea of respect for ancestors and older adults as a responsibility (Yeh & Bedford, 2020). Another possible reason is that 95% of the sample was female, aligning with previous research indicating that females in developed countries tend to exhibit lower ageist attitudes toward older adults than their male colleagues (Maximiano-Barreto et al., 2019).

The frequency of ageist behaviors among the participants in this study was .39. The negative ageist behavior frequency of .34 found in this study was higher than that for Korean health care professionals (Lee et al., 2020) and lower than that for Jordanian nurses (Rababa et al., 2020). Notably, the highest and second-highest scoring negative ageist behavior items in this study were “talk louder or slower to old people because of their age” and “use simple words when talking to old people”. These items may reflect that the participants often encountered older adults with hearing, cognitive, or physical impairments in their practice and, as a result, may reactively speak loudly or use simple words during interactions with older patients. These behaviors are generally viewed kindly by Taiwanese society rather than as reflecting discrimination. Furthermore, nurses in this study faced increased work pressure and occupational burnout due to the coronavirus disease 2019 (COVID-19) pandemic, driven by increased elderly mortality rates and health care resource shortages (Rababa et al., 2022). These factors may help explain the higher scores for negative ageist behavior found in this study.

The positive ageist behavior frequency of .51 found in this study is consistent with multiple prior research findings (Rababa et al., 2021; Rababa & Hammouri, 2021), and slightly higher than the frequency found among health care professionals and medical students in Korea (Lee et al., 2020). Interestingly, the frequency of positive ageist behaviors is higher than that of negative ageist behaviors in most studies. In this study, the top reported positive ageist behavior item was “hold doors open for old people because of their age”, followed by “When I find out an old person’s age, I may say, ‘You don’t look that old.’” A possible reason for this result is that societies, particularly those in East Asia influenced by Confucianism, tend to perceive these behaviors as polite and considerate towards older adults. However, this finding conflicts with the concept of positive ageist behaviors represented in the ROPE scale. Cary et al. (2017) reported that the seemingly warm and protective actions collectively defined as positive ageist behaviors are actually grounded in stereotypes and prejudices (collectively termed “ambivalent ageism”) that encapsulate both benevolence and hostility. As ambivalent ageism is frequently observed in traditional Chinese cultural settings (Tasdemir, 2020), future studies are needed to clarify whether ROPE scale items identify discrimination against or respect for older adults in East Asian societies. Furthermore, additional research is required to evaluate the suitability of the ROPE scale items due to its low Cronbach’s alpha for positive ageist behaviors in this and prior studies.

In this study, significant positive correlations were observed between age and, respectively, ageist behaviors, positive ageist behaviors, and negative ageist behaviors. Also, age was identified as a predictor of ageist behaviors among the participants. Bardach and Rowles (2012) found that older nurses had limited exposure to specialized training in gerontological care during their nursing education. Moreover, the demanding and fast-paced environment in acute settings may increase the risk and incidence of work-related burnout due to age-related declines in physical strength (Hatch et al., 2018), which may impact patient care quality and nurse behaviors toward their patients.

The results of this study also revealed significant differences in ageist behaviors across different hospital unit types, with the post hoc analysis results showing the participants working in ORs or CCUs had higher ageist behavior and negative ageist behavior frequencies than their peers in MSUs. However, no significant differences were found for positive ageist behaviors across hospital unit types. According to Hunt et al. (2020), the choice of specialty in nurses is often influenced by their interests and traits, which may help explain these findings. Also, Bulut and Cilingir (2016) mentioned that OR nurses may negatively perceive older patients due to communication barriers and view the care needs of these patients as excessive and challenging. Furthermore, the findings of this study identified working in CCUs as a predictor of ageist behaviors. One possible reason is that, during COVID-19, the high mortality rate among older adults with severe cases of the disease may have negatively affected the opinions of health care professionals toward older patients, resulting in more ageist behaviors (Rababa et al., 2022). Another reason may be that, in the emergency department’s fast-paced and task-oriented environment, nurses find it difficult to provide comprehensive care to older patients. This situation may lead ER nurses being more reluctant to care for older adults and developing negative stereotypes about them.

In this study, the RNs had a lower frequency of negative ageist behaviors than the licensed professional nurses (LPNs). In 2013, Taiwan discontinued the national licensing examination for LPNs, whose shorter education programs typically placed minimal focus on geriatric care (Lee et al., 2014). The findings of this study also revealed a positive relationship between job satisfaction and the frequency of positive ageist behaviors. Similarly, Hakami et al. (2020) observed that satisfied nurses are more engaged with patients in nursing practice, providing a reasonable explanation that nurses may display actions such as elderspeak that appear positive yet discriminatory during caring activities, as such behaviors in the Taiwan context are generally considered to be part of showing empathy towards older adults.

Also in this study, willingness to care for older adults was found to correlate positively with positive ageist behaviors. This correlation may reflect that a higher level of willingness to care for older people reflects greater attention to the needs and health of older adults. Consequently, nurses may unconsciously display positive ageist behaviors, for example, complimenting older adults for not looking that old despite their age, even though these behaviors may ultimately harm older adults (Marquet et al., 2019). Moreover, the participants who had taken age-related courses at school reported lower frequencies of both ageist behaviors and negative ageist behaviors than those who had never taken these courses. Notably, this variable was also identified as a predictor of ageist behaviors. As the findings of most prior research suggest that knowledge can shape attitudes and behaviors, levels of ageism and the frequency of ageist behaviors in nurses and nursing students should decline as their knowledge of aging increases (Rababa et al., 2020; Rababa et al., 2021). Developing appropriate curricular content to promote the perception of aging as a natural biological phenomenon and increasing related knowledge are crucial. Nursing faculty members should add age-related courses to programs and, if such courses are already being taught, their content should be reviewed and updated if needed.

This study revealed that higher aging anxiety is associated with higher frequencies of both ageist behaviors and negative ageist behaviors. Although aging is an inevitable biological process, many view it negatively due to the gradual decline in physical abilities and increased frailty, resulting in high levels of aging anxiety and ageism toward older adults (Donizzetti, 2019). Individuals have an unconscious fear of inevitable death out of an instinctive sense of self-protection, and their anxiety arises when confronted with uncontrollable situations such as death (Barnett & Adams, 2018). Moreover, Van (2020) mentioned that people may reflect a higher level of ageism because of negative perceptions and the threat of growing old that compels them to distance themselves from older adults to maintain their self-value and ego. Older patients face a higher risk of mortality in clinical settings than younger patients, which may enhance nurses’ awareness of the reality of death and evoke inner anxiety, exacerbating ageist behaviors.

Finally, higher levels of ageism were found to relate to higher frequencies of ageist behaviors and negative ageist behaviors and to be a predictor of ageist behaviors, which aligns with previous research on this issue in nursing students (Even et al., 2020; Rababa et al., 2021). Based on cognitive dissonance theory (Harmon-Jones et al., 2022), a strong relationship exists between attitudes and behaviors. Positive attitudes usually drive corresponding actions, while inconsistencies between attitudes and behaviors can create cognitive conflict that leads individuals to adjust their attitudes or behaviors to resolve the resultant contradictions, potentially explaining the path by which ageism influences ageist behaviors in nurses.

### Limitations of the Study

This study was affected by several limitations. As this was a cross-sectional study, changes in attitudes and behaviors could not be measured. Longitudinal studies should be conducted in the future to overcome this limitation. Second, this study recruited nurses from three hospitals in northern Taiwan only. Thus, the ability to generalize the findings to nurses nationwide and to other national settings is limited. Third, although the ROPE scale is a common tool for measuring individual ageist behaviors, certain items related to positive ageist behaviors are perceived as culturally appropriate behaviors in Taiwan. Thus, future research is needed to clarify this issue. Fourth, ageist behavior measurements relied on the retrospective recall of the participants, which may have introduced recall bias due to subjective and emotional factors.

### Conclusions

Ageism and ageist behaviors exist among hospital nurses. Based on the findings, significant predictors of ageist behaviors include age, working unit, having taken age-related courses at school, and ageism. Hospital nurses should be aware of their ageism and ageist behaviors and actively use bracketing techniques to prevent these biases from impacting their caregiving processes. In addition, future research is recommended to explore positive ageist behaviors to clarify the status of these behaviors in Asian cultures as either discriminatory or appropriate expressions of caring and respect. As many countries face nursing shortages and nurses may be floated to other units, nursing managers should develop training courses for nurses working in ORs and ICUs to cultivate proper behaviors towards older patients. Finally, nursing educators should design appropriate age-related courses to enhance students’ awareness of ageist behaviors.
